# MicroRNA‐551b‐3p inhibits tumour growth of human cholangiocarcinoma by targeting Cyclin D1

**DOI:** 10.1111/jcmm.14312

**Published:** 2019-06-14

**Authors:** Weiping Chang, Yuan Wang, WenZhi Li, Lei Shi, Zhimin Geng

**Affiliations:** ^1^ Department of Hepatobiliary Surgery The First Affiliated Hospital of Xi'an Jiaotong University Xi'an China; ^2^ Department of General Surgery The First Affiliated Hospital of Xi'an Medical University Xi'an China; ^3^ Department of Infectious Diseases The Second Affiliated Hospital of Xi'an Jiaotong University Xi'an China; ^4^ Chang'an District Hospital The First Affiliated Hospital of Xi'an Jiaotong University Xi'an China

**Keywords:** cholangiocarcinoma, Cyclin D1, microRNA‐551b‐3p, tumour growth

## Abstract

MicroRNAs (miRNAs) are powerful regulators in the tumorigenesis of cholangiocarcinoma (CCA). Previous studies report that miR‐551b‐3p acts as an oncogenic factor in ovarian cancer, but plays a tumour suppressive role in gastric cancer. However, the expression pattern and potential function of miR‐551b‐3p were still unclear in CCA. Therefore, this study aimed to explore the expression of miR‐551b‐3p and its role as well as molecular mechanism in CCA. Analysis of TCGA dataset suggested that miR‐551b‐3p was under‐expressed in CCA tissues compared to normal bile duct tissues. Furthermore, our data confirmed the decreased levels of miR‐551b‐3p in CCA samples and cell lines. Interestingly, TCGA data suggested that low miR‐551b‐3p level indicated reduced overall survival of CCA patients. Gain‐ and loss‐of‐function experiments found that miR‐551b‐3p inhibited the proliferation, G1‐S phase transition and induced apoptosis of CCA cells. In vivo experiments revealed that ectopic expression of miR‐551b‐3p inhibited tumour growth of CCA in mice. Further investigation demonstrated that miR‐551b‐3p directly bond to the 3′‐UTR of Cyclin D1 (CCND1) mRNA and negatively regulated the abundance of CCND1 in CCA cells. An inverse correlation between miR‐551b‐3p expression and the level of CCND1 mRNA was detected in CCA tissues from TCGA dataset. Notably, CCND1 knockdown showed similar effects to miR‐551b‐3p overexpression in HuCCT‐1 cells. CCND1 restoration rescued miR‐551b‐3p‐induced inhibition of proliferation, G1 phase arrest and apoptosis in HuCCT‐1 cells. In summary, miR‐551b‐3p inhibits the expression of CCND1 to suppress CCA cell proliferation and induce apoptosis, which may provide a theoretical basis for improving CCA treatment.

## 1 INTRODUCTION

Cholangiocarcinoma (CCA) is a common and aggressive malignancy with rising morbidity and mortality.^1^ CCA is divided into two subtypes (extrahepatic and intrahepatic) according to the tumour location. Lacking early diagnosis, refractory nature and aggressiveness of CCA lead to poor prognosis of patients. Though several treatment options and therapeutic targets for CCA have emerged, the survival rate of CCA patients is limitedly improved.^2^ Thus, it is still urgent to explore more detailed mechanisms involved in regulating and influencing CCA to achieve better clinical outcome.

MicroRNAs (miRNAs), important regulators of gene expression at post‐transcriptional level, exert their functions by directly binding to 3′‐untranslated region (3′‐UTR) of mRNAs, and subsequently regulate numerous biological and pathological processes, such as cell cycle progression, cell proliferation, apoptosis and differentiation. Increasing researches have revealed that aberrant miRNA levels are confirmed in various types of human cancer including CCA and play critical roles in the tumorigenesis and metastasis.^3^, ^4^, ^5^, ^6^ The levels of miR‐433 and miR‐22 are decreased in CCA, and forced expression of them lead to ciliary restoration and suppresses the malignant phenotype of cancer cells by targeting histone deacetylase 6 (HDAC6).^7^ miR‐21 is highly expressed in CCAs and impairs the sensitivity of cancer cells to heat shock protein 90 (HSP90) via repressing DnaJ heat shock protein family (Hsp40) member B5 (DNAJB5).^8^ Our previous study finds that miR‐125b‐5p is under‐expressed in CCA tissues and suppresses cell cycle progression and cell proliferation by targeting sirtuin7 (SIRT7).^9^ miR‐551b‐3p is recently recognized as a cancer‐associated miRNA in several human cancers. miR‐551b‐3p expression is markedly up‐regulated in papillary thyroid carcinoma (PTC) as compared with that in normal thyroid tissue.^10^ miR‐551b‐3p functions as an oncogenic factor via enhancing chemoresistance, invasion and proliferation of ovarian cancer (OVCa) by targeting forkhead box O3 (FOXO3) and tripartite motif containing 31 (TRIM31).^11^ Interestingly, the expression level of miR‐551b‐3p is reduced in gastric cancer and its mimics suppresses invasiveness, metastasis and epithelial‐mesenchymal transition of tumour cells by targeting erb‐b2 receptor tyrosine kinase 4 (ERBB4).^12^ Recently, a study reports that miR‐551b‐3p is reversely regulated by long non‐coding RNA (lncRNA) SMARCC2 and mediates the tumour promoting role of SMARCC2 through regulating transmembrane serine protease 4 (TMPRSS4) in gastric cancer.^13^ However, the functional role of miR‐551b‐3p and its underlying molecular mechanism in the tumorigenesis of CCA remain largely unclear.

This study explored the expression, functional role and potential mechanism of miR‐551b‐3p in CCA. Overexpression of miR‐551b‐3p inhibited CCA cell proliferation and resulted in cell cycle arrest at G1 phase and apoptosis by targeting Cyclin D1 (CCND1), which might benefit for finding new treatment modalities to improve prevention and treatment of CCA.

## 2 MATERIALS AND METHODS

### 2.1 Patients and tissue samples

The current research was approved by the Research Ethics Committee of 1st Affiliated Hospital of Xi'an Medical University, Xi'an, China. This retrospective study enrolled a total of 15 patients with primary CCA who underwent a curative liver resection in our hospital. The specimens were pathologically diagnosed as CCA, and no patient received any pre‐operative treatments. CCA tissues and 15 samples of normal bile duct tissue from patients with benign biliary disease were obtained for quantitative real‐time PCR (qRT‐PCR). All patients have signed the informed consent.

### 2.2 CCA cell lines

HuCCT‐1, RBE, HCCC‐9810, QBC939 cell lines and the human intrahepatic biliary epithelial cell line (HiBEC) were maintained in our laboratory.^9^ Cells were cultured in high‐glucose DMEM (Gibco, Thermo Fisher Scientific, Waltham, MA, USA) added with 10% FBS (Gibco), 1% penicillin (Gibco) and 100 μg/mL streptomycin (Gibco) in a humidified incubator containing 5% CO_2_ at 37°C.

### 2.3 Cell transfection

Vectors mediated miR‐551b‐3p and miR‐551b‐3p inhibitors (anti‐miR‐55b‐3p) as well as matched negative controls were obtained from GeneCopoeia (Guangzhou, China). pcDNA3.1‐CCND1, CCND1 siRNA (5′‐GGAGAACAAACAGAUCAUCTT‐3′; 5′‐GAUGAUCUGUUUGUUCUCCTC‐3′) and non‐targeting (NT, 5′‐CUUACGCUGAGUACUUCGATT‐3′; 5′‐UCGAAGUACUCAGCGUAAGTT‐3′) siRNA were purchased from GenePharma (Shanghai, China). Cell transfection was performed with Lipofectamine^®^ 2000 Reagent (Invitrogen, Carlsbad, CA, USA) following the manufacturer's protocols.

### 2.4 RNA extraction and qRT‐PCR

Total RNA samples were isolated from the collected tissues and cells, respectively. RNA extraction was performed with Trizol reagent (Invitrogen) according to the manufacturer's protocols. Then the RNA sample was reversely transcribed to cDNA using a TIANScript RT Kit (Tiangen Biotech, Beijing, China). qRT‐PCR was carried out using a SYBR Green PCR Kit (Takara, Shiga, Japan) on Applied Biosystems StepOne‐Plus Real‐Time PCR System (Applied Biosystems, Foster City, CA, USA). GAPDH and U6 were employed as internal controls for CCND1 mRNA and miR‐551b‐3p, respectively. qRT‐PCR primers for U6 (HmiRQP9001) and hsa‐miR‐551b‐3p (HmiRQP0651) were obtained from Genecopoeia. CCND1 primers: forward 5′‐CGT GGC CTC TAA GAT GAA GG‐3′ and reverse 5′‐CTG GCA TTT TGG AGA GGA AG‐3′. GAPDH primers: forward 5′‐TCA GTG GTG GAC CTG ACC TG‐3′ and reverse 5′‐TGC TGT AGC CAA ATT CGT TG‐3′. U6 primers: RT 5′‐AAC GCT TCA CGA ATT TGC GT‐3′, forward 5′‐GCT TCG GCA GCA CAT ATA CTA AAA T‐3′ and reverse 5′‐CGC TTC ACG AAT TTG CGT GTC AT‐3′. Has‐miR‐551b‐3p primers: RT 5′‐GTC GTA TCC AGT GCA GGG TCC GAG GTA TTC GCA CTG GAT ACG ACC TGA AA‐3′, forward 5′‐GAT ATG CGA CCC ATA CTT GG‐3′ and reverse 5′‐GTG CAG GGT CCG AGG T‐3′.

### 2.5 Cell proliferation analysis

Cell proliferation was detected using Cell Counting Kit‐8 (CCK‐8) and EdU assay. CCK‐8 solution (10 μL; Dojindo Molecular Technologies, Inc., Kumamoto, Japan) and EdU (50 μmol/L; RiboBio Biotechnology, Guangzhou, China) were used for these assays as previously described.^9^

### 2.6 Cell cycle distribution analysis

Briefly, cells were collected and fixed using 70% ethanol. After washing with PBS and subsequently washing with stain buffer, 1 × 10^6^ cells were resuspended in 0.5 mL of PI/RNase Staining Buffer (BD Biosciences, San Jose, CA, USA), and cells were incubated for 15 minutes at room temperature (RT), protected from light. A FACSCanto II flow cytometer (BD Biosciences) was used to analyse cell cycle distribution.

### 2.7 Apoptosis analysis

For apoptosis analysis, the PE Annexin V Apoptosis Detection Kit I (BD Biosciences) was used following the manufacturer's protocols. Cells were harvested and washed with pre‐cold PBS buffer twice. Then, 5 μL of PE Annexin V and 5 μL of 7‐AAD solution were added to each sample, and cells were incubated for 15 minutes at RT. FACSCanto II flow cytometer was used to measure the cell apoptosis.

### 2.8 In vivo tumour xenograft experiments

*In vivo* tumour xenograft experiments were performed with 10 BALB/c nude mice. In brief, 5 × 10^6^ HuCCT‐1 cells were implanted into the left flank of mice via subcutaneous injection. Tumour sizes were calculated using a vernier calliper as follows: tumour volume (mm^3^) = (L × W2)/2, where L = long axis and W = short axis. All subcutaneous tumour tissues were harvested 3 weeks later, and fixed with 4% paraformaldehyde, embedded, resected and stained with Ki‐67 to evaluate proliferation.

### 2.9 Dual luciferase reporter gene assay

Wild‐type (wt) 3′‐UTR of CCND1 and mutant type (mt) 3′‐UTR of CCND1 were sub‐cloned into PmirGLO plasmid (Promega, Madison, WI, USA). pmirGLO‐CCND1‐wt/‐mt and miR‐551b‐3p mimics/inhibitors were co‐transfected into HuCCT‐1 cells. The fluorescence intensity was detected by a dual‐Luciferase reporter assay system (Promega) after 24 hours of transfection.

### 2.10 Western blot assay

Cholangiocarcinoma cells were lysed by RIPA buffer (Beyotime, Jiangsu, China). The concentration of total protein was measured by BCA Assay Kit (Beyotime). Equal amount of proteins was separated by 10% SDS‐PAGE gel and then transferred onto PVDF membrane (Millipore, Bedford, MA, USA). The membranes were blocked in 5% skimmed milk at 4°C and then incubated with CCND1 primary antibody (Abcam, Cambridge, MA, USA) and β‐actin primary antibody (Abcam) overnight at 4°C. Next, the membranes were incubated with HRP‐conjugated IgG secondary antibodies (Santa Cruz Biotechnology, Santa Cruz, CA, USA) for 1‐2 hours. Then, protein expression levels were detected by ECL (Millipore).

### 2.11 Statistical analysis

The statistical analyses were carried out using the software of GraphPad Prism version 6.0 (GraphPad Inc., San Diego, CA, USA). The Student's *t* test and ANOVA were used to compare the statistical differences between groups. A *P* < 0.05 was considered to indicate a significant difference.

## 3 RESULTS

### 3.1 miR‐551b‐3p is down‐regulated in CCA

**Figure 1:**
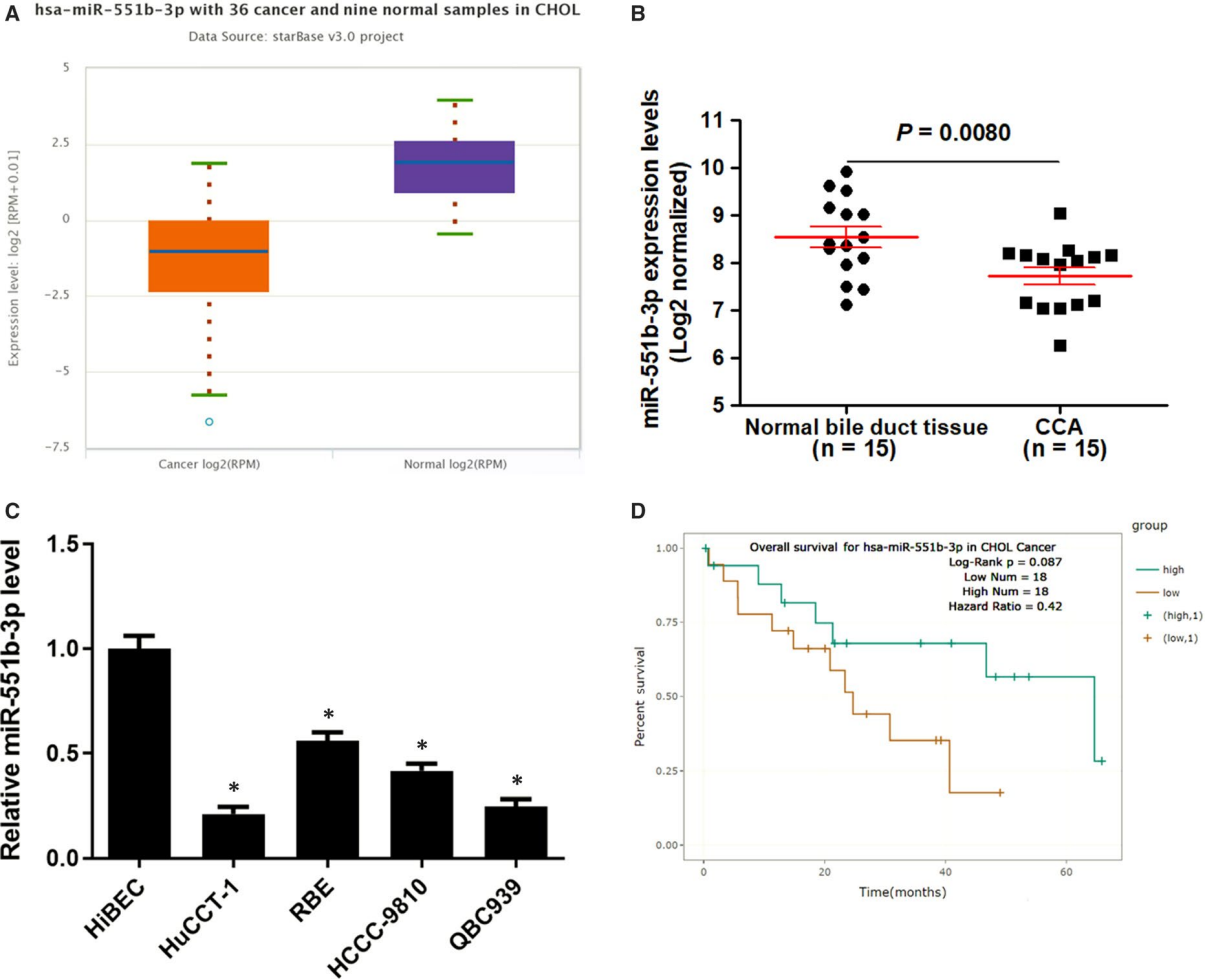
The expression of miR‐551b‐3p in CCA. A, CCA samples (n = 36) and normal bile duct tissues (n = 9) in TCGA dataset from starBase V3.0 platform showed that miR‐551b‐3p expression was significantly down‐regulated in tumour tissues. B, The expression of miR‐551b‐3p was detected in CCA tissues (n = 15) and normal bile duct tissues (n = 15) using qRT‐PCR. C, The levels of miR‐551b‐3p between CCA cell lines (HuCCT‐1, RBE, HCCC‐9810 and QBC939) and HiBEC cells were analysed by qRT‐PCR. *P < 0.05. C, TCGA data from starBase V3.0 platform indicated that CCA patients with low miR‐551b‐3p level had an obvious poorer overall survival compared to miR‐551b‐3p high‐expressing patients

We explored miR‐551b‐3p expression in TCGA Data Portal from starBase V3.0,^14^ the data discovered that the level of miR‐551b‐3p was down‐regulated in CCA samples compared to normal bile duct tissues (*P* = 0.00041, Figure 1A). Further analysis of miR‐551b‐3p expression in CCA samples from our hospital suggested that miR‐551b‐3p expression was reduced in CCA samples compared to normal bile duct tissues (*P* = 0.0080, Figure 1B), which was consistent with the result of TCGA dataset. The miR‐551b‐3p expression in normal HiBEC and CCA cell lines (HuCCT‐1, HCCC‐9810, RBE and QBC939) was measured by qRT‐PCR. The results indicated that miR‐551b‐3p expression in CCA cells was significantly lower than that in HiBEC cells (*P* < 0.05, Figure 1C). Interestingly, Pan‐Cancer Survival Analysis of microRNA Genes across 32 types of Cancers from starBase V3.0^14^ indicated that low miR‐551b‐3p level might predict poor overall survival of CCA patients (Figure 1D). Thus, our results suggested that miR‐551b‐3p might play a key role in CCA progression.

### 3.2 miR‐551b‐3p regulates the proliferation, cell cycle progression and apoptosis of CCA cells

**Figure 2:**
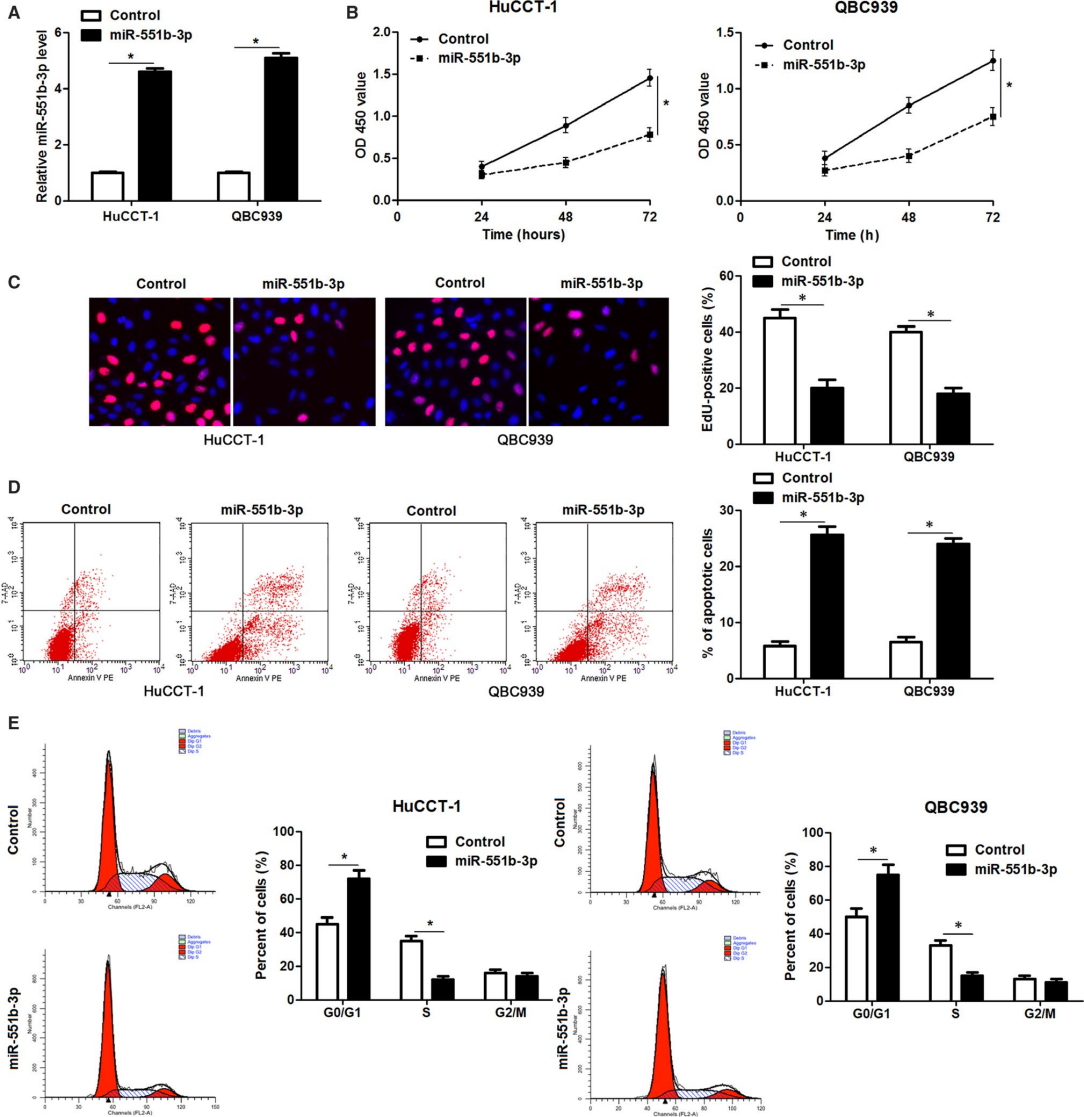
miR‐551b‐3p overexpression suppresses proliferation, cell cycle progression and induces apoptosis of CCA cells. A, Vectors containing miR‐551b‐3p or control mimics were transfected into HuCCT1 and QBC939 cells and detected by qRT‐PCR for miR‐551b‐3p expression. B and C, CCK‐8 and EdU assays demonstrated that miR‐551b‐3p overexpression inhibited CCA cell proliferation. D, miR‐551b‐3p overexpression induced the apoptosis of CCA cells as suggested by flow cytometry analysis. E, The G1 to S phase transition was blocked by miR‐551b‐3p overexpression in HuCCT1 and QBC939 cells. *P < 0.05

**Figure 3:**
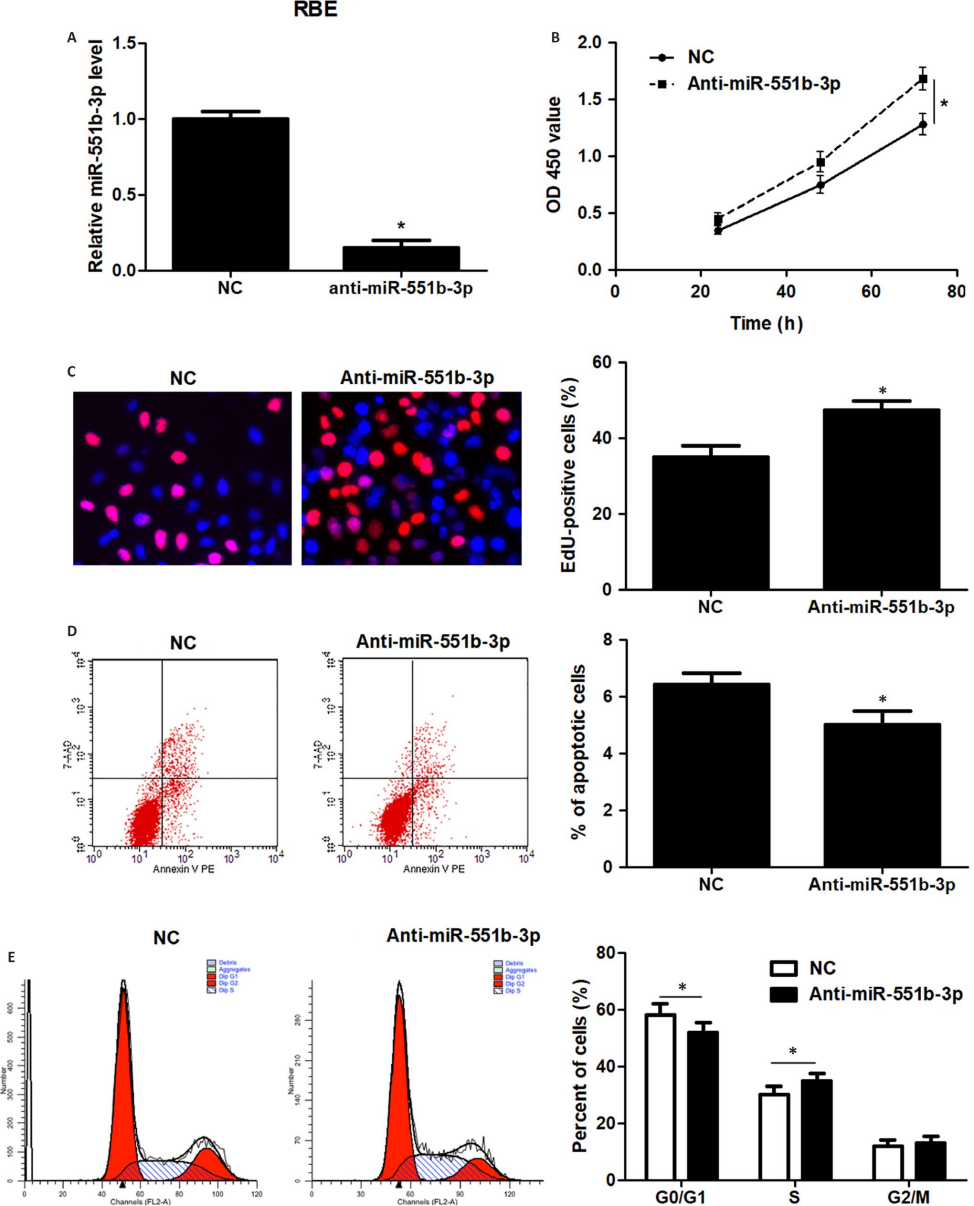
miR‐551b‐3p knockdown enhances proliferation, cell cycle progression and inhibits apoptosis of RBE cells. A, Vectors containing miR‐551b‐3p inhibitors or negative control (NC) were transduced into RBE cells and detected for miR‐551b‐3p expression using qRT‐PCR. B and C, CCK‐8 and EdU assays demonstrated that miR‐551b‐3p knockdown enhanced CCA cell proliferation. D, miR‐551b‐3p knockdown inhibited the apoptosis of RBE cells as suggested by flow cytometry analysis. E, miR‐551b‐3p knockdown facilitated the G1 to S phase transition in RBE cells. *P < 0.05

Further experiments were carried out to clarify the function of miR‐551b‐3p in CCA cells. Forced expression of miR‐551b‐3p was performed in HuCCT‐1 and QBC939 cells (*P* < 0.05, Figure 2A). CCK‐8 and EdU assays clarified that miR‐551b‐3p overexpression markedly restrained the proliferation of CCA cells (*P* < 0.05, Figure 2B and C). Flow cytometry measurement showed that the percentage of apoptotic CCA cells was markedly elevated by miR‐551b‐3p overexpression (*P* < 0.05, Figure 2D). miR‐551b‐3p overexpression led to cell cycle arrest at G1 phase in HuCCT‐1 and QBC939 cells (*P* < 0.05, Figure 2E). Conversely, miR‐551b‐3p knockdown evidently enhanced RBE cell proliferation and G1‐S phase transition, and reduced apoptosis in vitro (*P* < 0.05, Figure 3A‐E). These findings indicated that miR‐551b‐3p inhibited the progression of CCA by inducing growth arrest and apoptosis.

### 3.3 miR‐551b‐3p overexpression represses tumour growth of CCA in mice

**Figure 4:**
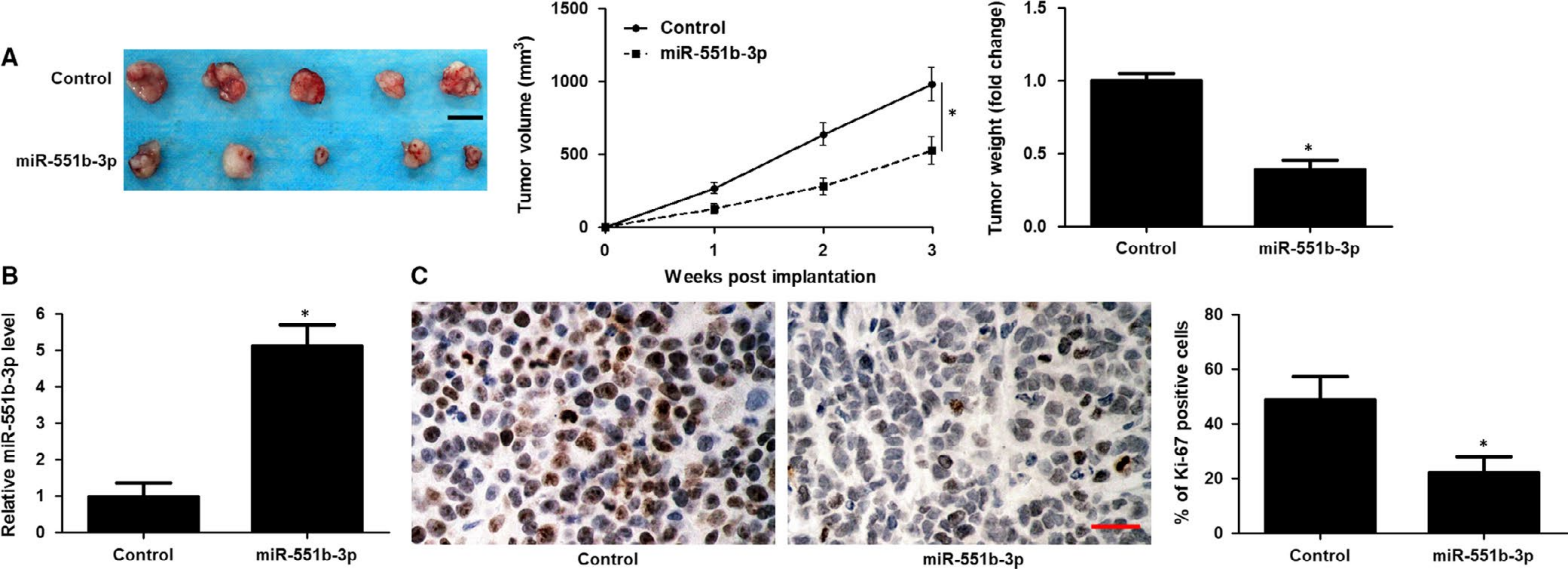
miR‐551b‐3p restoration restrains tumour growth of HuCCT‐1 cells. A, HuCCT‐1 cells that were transfected with vectors containing miR‐551b‐3p or control mimics were subcutaneously implanted into nude mice . Both tumour volume and tumour weight in miR‐551b‐3p group were obviously less than those in control group. B, The expression of miR‐551b‐3p in xenograft tumour tissues from miR‐551b‐3p group was significantly higher than that in control group. C, IHC analysis showed that strong staining of Ki‐67 was observed in most of tumour cells in control group, whereas only several tumour cells showed weak staining of Ki‐67 in miR‐551b‐3p group. *P < 0.05

To further confirm the anti‐tumour role of miR‐551b‐3p in vivo, a subcutaneous tumour formation model was constructed using HuCCT‐1 cells with or without miR‐551b‐3p overexpression. Notably, the tumour size and tumour weight in miR‐551b‐3p group were markedly less than those in control group (*P* < 0.05, Figure 4A). The expression of miR‐551b‐3p in xenograft tumour tissues from miR‐551b‐3p group was markedly higher than that in control group (*P* < 0.05, Figure 4B). Moreover, the rate of Ki‐67 positive tumour cells in miR‐551b‐3p group was prominently lower as compared with that in control group (*P* < 0.05, Figure 4C). Taken together, miR‐551b‐3p acted as a tumour suppressive factor in CCA.

### 3.4 CCND1 is a downstream target of miR‐551b‐3p

**Figure 5:**
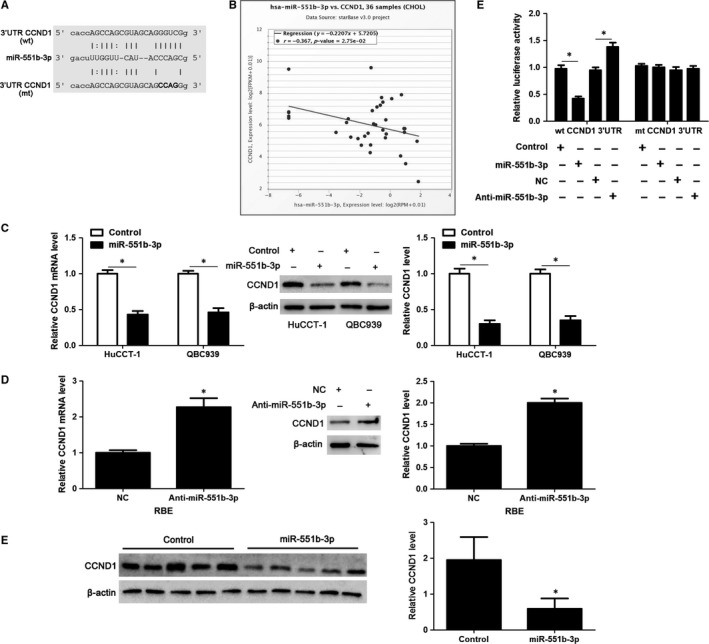
CCND1 is recognized as a target of miR‐551b‐3p in CCA cells. A, Bioinformatic analysis in starBase V3.0 platform indicated the potential binding sequences between miR‐551b‐3p and 3′UTR of CCND1. B, TCGA data from starBase V3.0 platform indicated that the expression of miR‐551b‐3p was negatively correlated with the level of CCND1 mRNA in CCA tissues. C, miR‐551b‐3p or control mimics were transfected into HuCCT‐1 and QBC939 cells and detected for CCND1 expression using qRT‐PCR and immunoblotting. D, miR‐551b‐3p inhibitors or negative control (NC) was transduced into RBE cells and detected for CCND1 expression using qRT‐PCR and immunoblotting. E, The expression of CCND1 protein in xenograft tumour tissues from miR‐551b‐3p group was significantly lower than that in control group. (F) Co‐transfection of miR‐551b‐3p mimics/inhibitors and wild‐type (wt) or mutated (mt) 3′UTR of CCND1 was performed in HuCCT‐1 cells and the relative luciferase activity was measured. *P < 0.05

According to the online analysis with bioinformatics database (starBase V3.0^14^), there was a specific binding region between the 3′UTR of CCND1 and miR‐551b‐3p, and CCND1 was predicted to be a potential target of miR‐55b‐3p (Figure 5A). Pan‐Cancer Co‐Expression Analysis for the miRNA‐target interactions across 32 types of cancers from starBase V3.0^14^ revealed that miR‐551b‐3p level was significantly inversely correlated with the level of CCND1 mRNA in CCA samples (*r* = −0.367, *P* = 0.0275, Figure 5B). Notably, miR‐551b‐3p overexpression markedly decreased the levels of CCND1 mRNA and protein in HuCCT‐1 and QBC939 cells (*P* < 0.05, Figure 5C). While, miR‐551b‐3p knockdown prominently increased the abundance of CCND1 in RBE cells (*P* < 0.05, Figure 5D). The expression of CCND1 protein in xenograft tumour tissues from miR‐551‐3p group was significantly lower than that in control group (*P* < 0.05, Figure 5E). Next, we found that the fluorescence intensity was decreased in cells co‐transfected with miR‐551b‐3p mimics and wt 3′UTR of CCND1, whereas enhanced in cells co‐transfected with miR‐551b‐3p inhibitors and wt 3′UTR of CCND1 (*P* < 0.05, Figure 5F). However, no noticeable difference was observed in fluorescence intensity of cells co‐transfected with miR‐551b‐3p mimics/inhibitors and mt 3′UTR of CCND1 (Figure 5F). These data displayed that CCND1 was a direct target of miR‐551b‐3p.

### 3.5 miR‐551b‐3p inhibits the growth of HuCCT‐1 cells through inhibiting CCND1

**Figure 6:**
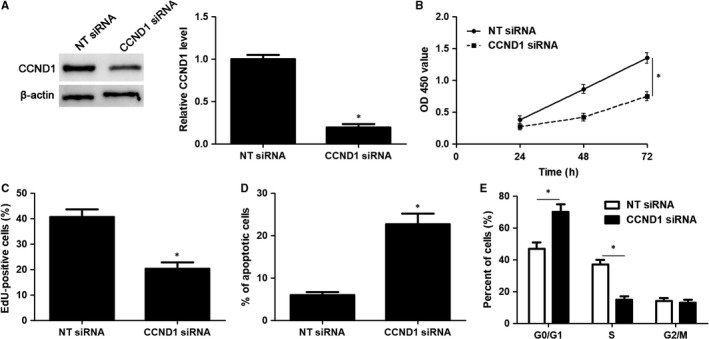
CCND1 knockdown suppresses the growth of HuCCT‐1 cells. A, CCND1 siRNA or non‐targeting (NT) siRNA was transfected into HuCCT‐1 cells and detected for CCND1 protein using immunoblotting. B, CCK‐8, (C) EdU, (D) flow cytometry apoptosis and (E) cell cycle analysis were carried out to detect the proliferation, apoptosis and cell cycle progression of HuCCT‐1 cells. *P < 0.05

**Figure 7:**
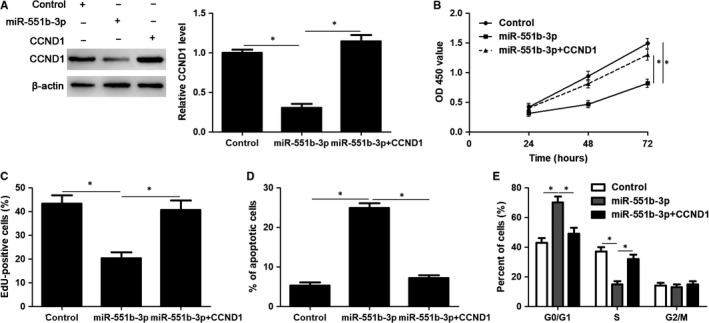
CCND1 restoration rescues miR‐551b‐3p induced growth arrest and apoptosis of HuCCT‐1 cells. A, CCND1 expression was rescued by transfecting vectors in miR‐551b‐3p overexpressing HuCCT‐1 cells as detected by immunoblotting. B, CCK‐8, (C) EdU, (D) flow cytometry apoptosis and (E) cell cycle analysis were carried out to detect the proliferation, apoptosis and cell cycle progression of HuCCT‐1 cells. CCND1 restoration abrogated the effects of miR‐551b‐3p on HuCCT‐1 cells. *P < 0.05

CCND1 knockdown was performed with a specific siRNA in HuCCT‐1 cells (*P* < 0.05, Figure 6A). Notably, CCND1 knockdown significantly induced inhibition of proliferation, G1 phase arrest and apoptosis in HuCCT‐1 cells (*P* < 0.05, Figure 6B‐E), which was consistent with the effects of miR‐551b‐3p overexpression. Accordingly, CCND1 expression was rescued by plasmid transfection in HuCCT‐1 cells with miR‐551b‐3p overexpression (*P* < 0.05, Figure 7A). Then, CCK‐8 and EdU assays demonstrated that CCND1 restoration significantly enhanced the proliferation of miR‐551b‐3p overexpressing HuCCT‐1 cells (*P* < 0.05, Figure 7B and C). CCND1 restoration rescued miR‐551b‐3p induced G1 phase arrest and apoptosis in HuCCT‐1 cells (*P* < 0.05, Figure 7D and E). These results indicated that miR‐551b‐3p suppressed CCND1 expression and thereby repressed tumour growth of CCA.

## 4 DISCUSSION

Despite many studies have demonstrated the critical role of miR‐551b‐3p in the pathogenesis of human cancers, the expression and functional role of miR‐551b‐3p in CCA remain unknown. Overexpression of miR‐551b‐3p is confirmed in PTC and OVCa,^10^, ^11^ and underexpression of miR‐551b‐3p is detected in gastric cancer.^12^, ^13^ This study confirmed that miR‐551b‐3p was under‐expressed in CCA tissues and cell lines. A recent study has revealed potential mechanism involved in aberrant expression of miR‐551b‐3p in gastric cancer. LncRNA SMARCC2 functions as a competing endogenous RNA (ceRNA) to inversely regulate miR‐551b‐3p abundance in gastric cancer.^13^ Furthermore, hypermethylation may be implicated in repressed expression of miR‐551b in breast cancer.^15^ Thus, it is worth to further investigate whether lncRNA or hypermethylation contributes to down‐regulation of miR‐551b‐3p expression in CCA. Interestingly, TCGA data indicated that lower expression of miR‐551b‐3p potentially predicted a poorer overall survival of CCA patients. Previous study demonstrates that low miR‐551b‐3p expression indicates a poor prognosis of patients with gastric cancer.^12^ Moreover, reduced miR‐551b level confers to shorter survival of patients with lung adenocarcinoma.^16^ Thereby, more CCA sample is required to further confirm the prognostic value of miR‐551b‐3p.

Increasing evidence has demonstrated that miRNAs are important drivers for tumour growth and metastasis of CCA. miR‐191 plays a tumour promoting role in CCA by facilitating cell invasion, migration and proliferation.^17^ Increased level of miR‐181c contributes to chemoresistance, proliferation and metastasis of CCA cells.^18^ Moreover, nanoparticles‐delivered miR‐210 inhibitors show markedly anti‐tumour effects on CCA cells.^19^ Functionally, miR‐551b‐3p facilitates tumour growth, migration and invasion via down‐regulating FOXO3 and TRIM31 in OVCa.^11^ Inversely, miR‐551b‐3p suppresses gastric cancer cell proliferation, migration and invasion through targeting ERBB4 and TMPRSS4.^12^, ^13^ In the current study, the overexpression of miR‐551b‐3p reduced CCND1 expression and inhibited the proliferation, G1‐S phase transition and induced apoptosis of CCA cells. Additionally, miR‐551b‐3p knockdown increased CCND1 expression and facilitated the proliferation, cell cycle progression and inhibited apoptosis of RBE cells. The expression of miR‐551b‐3p in RBE cells was also significantly lower than that in HiBEC cells. Thus, the loss‐of‐function study of miR‐551‐3p showed subtle but significant effects in RBE cells. In addition, we recognized CCND1 as a direct target of miR‐551b‐3p in CCA cells. CCND1 is an important G1‐S modulator to promote rapid progression from G1 to S phase and facilitates the proliferation of CCA cells. Elevated expression of CCND1 in CCA samples has been previously reported and its overexpression is correlated with poor clinical features and outcome of patients.^20^, ^21^ CCND1 is negatively regulated by several miRNAs including let‐7a, miR‐155, miR‐503 and miR‐302 in human cancers.^22^, ^23^, ^24^, ^25^, ^26^ Overexpression of miR‐155 and let‐7a lead to inhibition of proliferation, G1 phase arrest and apoptosis via repressing CCND1 in gastric cancer and lung adenocarcinoma, respectively.^22^, ^23^ miR‐503 inhibits tumour growth and G1/S transition by repressing CCND1 in endometrioid endometrial cancer.^26^ However, previous study reveals that miR‐302 suppresses the proliferation and migration of endometrial cancer cells, and leads to G2/M arrest by targeting CCND1 and CDK1,^25^ suggesting that other potential targets of miR‐302 may participate in regulating cell cycle progression. Here, we found that CCND1 knockdown showed similar effects with miR‐551b‐3p overexpression on CCA cells. And co‐transfection of CCND1 abrogated the tumour suppressive role of miR‐551b‐3p overexpression in CCA cells. These findings support that miR‐551b‐3p plays a tumour suppressive role in CCA, at least partly, via directly targeting CCND1.

In summary, we demonstrated for the first time that miR‐551b‐3p was low‐expressed in CCA. Functionally, miR‐551b‐3p inhibited cell proliferation, suppressed cell cycle progression and induced apoptosis by repressing CCND1 in CCA. Thus, miR‐551b‐3p may be a potential therapeutic target for CCA.

## 5 CONCLUSIONS

To conclude, we have demonstrated that down‐regulation of miR‐551b‐3p is a frequent event in CCA. miR‐551b‐3p underexpression is potentially associated with poor survival of CCA patients. miR‐551b‐3p inhibits CCA cell proliferation, suppressed cell cycle progression and induces apoptosis in vitro and in vivo. CCND1 is a novel downstream target of miR‐551b‐3p and mediates the tumour suppressive role of miR‐551b‐3p. Our results may provide a novel theoretical and experimental basis for the pathogenesis of CCA, and identify novel treatment targets.

## CONFLICT OF INTEREST

All authors declare no conflict of interest.

## AUTHOR CONTRIBUTION

Zhimin Geng conceived and designed the experiments; Weiping Chang, Yuan Wang and Wenzhi Li performed the experiments; Weiping Chang and Zhimin Geng analysed the data; Lei Shi contributed reagents/materials/analysis tools; Weiping Chang and Zhimin Geng wrote the paper. All authors read and approved the final manuscript.
